# T-Cell Epitope-Based SARS-CoV-2 DNA Vaccine Encoding an Antigen Fused with Type 1 Herpes Simplex Virus Glycoprotein D (gD)

**DOI:** 10.3390/v17091191

**Published:** 2025-08-30

**Authors:** Luana Raposo de Melo Moraes Aps, Aléxia Adrianne Venceslau-Carvalho, Carla Longo de Freitas, Bruna Felício Milazzotto Maldonado Porchia, Mariângela de Oliveira Silva, Lennon Ramos Pereira, Natiely Silva Sales, Guilherme Formoso Pelegrin, Ethiane Segabinazi, Karine Bitencourt Rodrigues, Jamile Ramos da Silva, Bianca da Silva Almeida, Jéssica Pires Farias, Maria Fernanda Castro-Amarante, Paola Marcella Camargo Minoprio, Luís Carlos de Souza Ferreira, Rúbens Prince dos Santos Alves

**Affiliations:** 1 Vaccine Development Laboratory, Microbiology Department, Biomedical Sciences Institute, University of São Paulo, São Paulo 05508-000, Brazil; luana.raposo@imunotera.com.br (L.R.d.M.M.A.); lcsf@usp.br (L.C.d.S.F.); 2 ImunoTera Soluções Terapêuticas Ltd.a., São Paulo 01452-922, Brazil; 3 Institut Pasteur de São Paulo, São Paulo 05508-020, Brazil

**Keywords:** polyepitope DNA vaccine, SARS-CoV-2, glycoprotein D (HSV-1), K18-hACE2 mouse model

## Abstract

Authorized SARS-CoV-2 vaccines elicit both antibody and T-cell responses; however, benchmark correlates and update decisions have largely emphasized neutralizing antibodies. Motivated by the complementary role of cellular immunity, we designed a prototype polyepitope DNA vaccine encoding conserved human and mouse T-cell epitopes from non-structural proteins of the original strain SARS-CoV-2 lineage. Epitope selection was guided by in silico predictions for common HLA class I alleles in the Brazilian population and the mouse H-2Kb haplotype. To enhance immunogenicity, the polyepitope sequences were fused to glycoprotein D (gD) from Herpes Simplex Virus 1 (HSV-1), an immune activator of dendritic cells (DCs), leading to enhanced activation of antigen-specific T-cell responses. Mice were immunized with two doses of the electroporated DNA vaccine encoding the gD-fused polyepitope, which induced robust interferon-gamma– and tumor necrosis factor-alpha–producing T cell responses compared to control mice. In addition, K18-hACE2 transgenic mice showed protection against intranasal challenge with the original SARS-CoV-2 strain, with reduced clinical symptoms, less weight loss, and decreased viral burden in both lung and brain tissues. The results experimentally confirm the protective role of T cells in vaccine-induced protection against SARS-CoV-2 and open perspectives for the development of universal anti-coronavirus vaccines.

## 1. Introduction

Since its identification in December 2019, the severe acute respiratory syndrome coronavirus 2 (SARS-CoV-2) has resulted in over 770 million confirmed cases and more than 7 million deaths worldwide [1]. COVID-19 presents a broad clinical spectrum, ranging from asymptomatic infection to severe respiratory distress and multi-organ dysfunction, and continues to pose significant public health challenges globally [2,3]. The unprecedented speed, design, production, and testing of anti-COVID-19 vaccines marked an important milestone in the history of vaccine development. This represented an essential step in the rapid control of the pandemic [4,5]. Another unique aspect of the anti-SARS-CoV-2 vaccine development was the use of different technological platforms, including, for the first time, mRNA and adenovirus-vector-based vaccines [5]. All clinically approved anti-SARS-CoV-2 vaccines rely on the induction of virus-neutralizing antibodies, which remain the primary correlate of protection and the key requirement for approval of new vaccine formulations [6,7,8,9].

T lymphocytes play a crucial role in controlling viral infections and establishing long-term immunological memory [10,11]. Clinical studies have shown that individuals mounting robust CD4^+^ and CD8^+^ T-cell responses experience better clinical outcomes and a lower risk of severe COVID-19, even in the absence of neutralizing antibodies. At the molecular level, T-cell epitopes have been mapped across several SARS-CoV-2 proteins—including spike (S), nucleocapsid (N), membrane (M), and ORF regions—with many of these epitopes showing high conservation across multiple viral lineages, including variants of concern (VOCs), such as Delta and Omicron [12,13]. Numerous preclinical studies have proposed polyepitope T-cell-centric vaccine designs that incorporate conserved fragments from across the SARS-CoV-2 proteome [14,15,16,17].

Building on this reasoning, we revisited the rationale and searched for experimental evidence that T-cell–epitope-centric vaccines are indeed an alternative for developing anti-coronavirus vaccines. For this purpose, we generated a DNA vaccine encoding an antigen designed through a thorough immune engineering approach, containing conserved T-cell epitopes across multiple non-structural SARS-CoV-2 proteins, for both common HLA class I alleles in the Brazilian population and the mouse H-2Kb haplotype. We also tested the adjuvant effects of type 1 Herpes Simplex Virus glycoprotein (HSV-1 gD) by generating a genetically fused chimeric protein, based on previous studies demonstrating the potent impact of gD on activating T-cell responses [18,19,20]. In vivo evidence indicates that immunization with the vaccine encoding the gD-fused polyepitope antigen induces robust and durable T-cell responses, which correlate with improved disease outcomes and partial protection against SARS-CoV-2 challenge in a murine model. The results confirm the protective role of T cells in immune defense against SARS-CoV-2 and support the future development of T-cell-based, pan-variant coronavirus vaccines.

## 2. Materials and Methods

### 2.1. Mice and Tissue Collection

For this study, C57BL/6 mice and B6.Cg-Tg(K18-ACE2)2Prlmn/J (The Jackson Laboratory, Bar Harbor, Maine), transgenic mice that express the human ACE2 receptor, were used. The sex ratio for all experiments was approximately 1:1, and all experiments commenced when the mice were 5 to 7 weeks old. For tissue collection, the mice were euthanized by CO_2_ inhalation. SARS-CoV-2 infections were performed in our high containment facility ABSL3 Laboratory at the Institute of Biomedical Science (ICB—University of São Paulo, São Paulo 05508-000, Brazil) and the Institute Pasteur of São Paulo. All experiments were performed in strict accordance with recommendations outlined in the Ethical Principles of Animal Experimentation established by the Brazilian College of Animal Experimentation (CEUA Nº 4733050620, CEUA Nº 7040290920).

### 2.2. Plasmid Design

To identify the target sequences within the non-structural proteins of the SARS-CoV-2 original strain (Wuhan), we conducted an associative analysis between the predicted epitopes and the HLA class I molecules that are most common in the Brazilian population. The MHC H-2 Kb (C57BL/6) binding epitopes were predicted for the selected regions. The allele frequency of HLA class I in the Brazilian population was obtained from the NCBI database https://www.ncbi.nlm.nih.gov/projects/gv/mhc/ihwg.cgi (accessed on 12 March 2020). Alleles that were present in at least 3% of the Brazilian population were selected (HLA: C*02:02, A*32:01, A*68:02, A*01:01, B*08:01, A*65:02, B*51:01, B*35:01) [21]. Epitopes within the amino acid sequence of the replicase polyprotein 1ab (PR1ab) were predicted using the Immune Epitope Database (IEDB) analysis resource http://tools.immuneepitope.org/mhci/ (assessed on 12 March 2020), following the recommended method. The predicted epitopes within PR1ab were ranked by their percentage values, and for each HLA molecule considered, the top five with a percentile lower than 0.5 were selected. Epitopes with lengths of 8, 9, 10, and 11 amino acids for HLA Class I were considered. After associative analysis, we identified two regions in PR1ab that concentrate most of the target sequences for humans and mice (NSa and NSb). The sequences of both regions, along with the predicted tandem peptides, were incorporated into the pcDNA3.1 vaccine vector as fusions to full-length herpesvirus glycoprotein D (gD) [20], with a Kozak sequence under the control of the CMV promoter. The plasmids—pgDNSa (~9.99 kb), pgDNSb (~9.77 kb), pgPOLYEP (~8.63 kb), and pPOLYEP (~7.44 kb)—and the corresponding peptides (reversed-phase HPLC, ≥95% purity) were obtained commercially from GenScript (Piscataway, NJ, USA).

### 2.3. In Vitro Expression of the Vaccine Antigens

As described previously [22], HEK293-T cells were seeded at 1 × 10^5^ cells per well in 24-well plates in DMEM supplemented with 10% fetal bovine serum (FBS) and allowed to grow to 70–80% confluence (37 °C, 5% CO_2_). Cells were transfected with the recombinant plasmids pgDNSa, pgDNSb, pgPOLYEP, and empty pCDNA3.1 as a control using Lipofectamine 3000 (Thermo Fisher Scientific, Waltham, MA, USA), following the manufacturer’s instructions. After 24 h, the cells were rinsed three times with PBS, and protein expression was assessed using immunofluorescence microscopy.

To detect target protein expression in transfected HEK293-T cells, the cells were fixed using a 4% paraformaldehyde (PFA) solution in phosphate-buffered saline (PBS) for 15 min at room temperature. The cells were then permeabilized with 0.1% Triton X-100 in PBS for 10 min at room temperature. The monolayer was washed twice and blocked with 2% BSA in PBS for 30 min at room temperature. It was then immunolabeled with mouse anti-gD antibody, previously diluted in blocking solution. After one hour, the cells were washed three times with PBS and labeled for 45 min at room temperature with Alexa Fluor 488-conjugated (Invitrogen, Carlsbad, CA, USA) anti-mouse/human IgG (Invitrogen, Carlsbad, CA, USA), under agitation. The cells were rewashed and incubated for 20 min at room temperature with Hoechst 33,342 (Life Technologies, Carlsbad, CA, USA) diluted 1/500 in PBS. Finally, after another wash cycle, images were captured using an Evos FL (Thermo Fisher Scientific, Waltham, MA, USA) with a 100× and 200× dry objective.

### 2.4. Virus Propagation and Titration

SARS-CoV-2 (severe acute respiratory syndrome coronavirus 2) isolate lineage B.1.1.28.2 (related to the original Wuhan SARS-CoV-2 strain and stored in the virus collection of the Institut Pasteur de São Paulo) was used throughout the study. This strain was cultured in Vero cells (ATCC, Manassas, VA, USA, CCL81) in Dulbecco’s Modified Eagle’s Medium (Corning, NY, USA) supplemented with 2% fetal bovine serum (FBS) and 1% penicillin-streptomycin for 3 days. It was then harvested and titrated using a plaque assay. Briefly, 10-fold serially diluted viral supernatants were added to confluent Vero E6 cells in 24-well plates (1 × 10^5^ cells/well) for 2 h at 37 °C, the supernatants were removed, 1% carboxymethylcellulose medium was added to each well, and the plates were incubated for 3 days. Cells were then fixed and stained with Naphthol Blue Black (Sigma-Aldrich, St. Louis, MO, USA) dissolved in sodium acetate–acetic acid (0.1% amido black solution [*w*/*w*] with 5.4% acetic acid, 0.7% sodium acetate) for 30 min at room temperature. Plaque-forming units (PFUs) were counted.

### 2.5. Vaccination and Infection

C57BL/6 mice (*n* = 8) and B6.Cg-Tg (K18-ACE2)2Prlmn/J transgenic mice (*n* = 8) were vaccinated intramuscularly (IM) in the quadriceps using electroporation with a minimally invasive device, applying 2 electrical pulses of 45 V each, with a duration interval of 450 ms (pulses that form pores in the cell membrane), and 4 pulses of 20 V each, with a duration of 450 ms (transfer pulses) (NEPA21 Super Electroporator, NepaGene, Japan) [20]. They received 50 μg of pgDNSa, pgDNSb, pgPOLYEP, pPOLYEP, and pCDNA3.1 vaccines and were boosted 14 days later using the same method [22]. Mice were infected intranasally (IN) with 5 × 10^4^ PFU of the SARS-CoV B.1.1.28 (GenBank: MW441769.1) as described elsewhere [22,23]. Body weight and clinical disease scores were recorded daily after the virus challenge, based on the following symptoms: piloerection, kyphosis, paralysis, and moribund condition.

### 2.6. Flow Cytometry and Intracellular Cytokine Staining (ICS) Analysis

To generate single-cell splenocytes, spleens from mice immunized 14 days after the last vaccine dose were removed aseptically and macerated using cell strainers (BD Biosciences, San Jose, CA, USA) to obtain a cell suspension. Red blood cells were lysed with ACK Lysis Buffer (BioSource International, Camarillo, CA, USA) for 5 min on ice and then centrifuged for 5 min at 1200 rpm at 4 °C. After an additional wash with RPMI, cells were placed in 96-well round-bottom plates at 2 × 10^6^ cells per well in complete RPMI and stimulated for 2 h (37 °C, 5% CO_2_) in the presence of antigen-specific peptides (pep4- VSFCYMHHM; pep5- VAYFNMVYM), followed by the addition of Brefeldin A (GolgiPlug; BD Biosciences, San Jose, CA, USA) for 4 h. Positive and negative controls were stimulated for the same duration with Ionomycin (1 µg/mL) and Brefeldin A (1 µg/mL), or no stimulant, respectively. At the end of the 4-h incubation, cells were stained with a combination of fluorophore-conjugated antibodies against mouse CD3e (clone 17A2, BioLegend, San Diego, CA, USA), CD8α (clone 53–6.7, BioLegend, San Diego, CA, USA), CD4 (clone GK1.5, BioLegend, San Diego, CA, USA), CD11a (clone M17/4, eBioscience, San Diego, CA, USA), and CD49d (clone R1-2, eBioscience, San Diego, CA, USA). Cells were then fixed and permeabilized with Cytofix/Cytoperm and stained with fluorophore-conjugated antibodies against mouse IFNγ (clone XMG1.2, Biolegend, San Diego, CA, USA) and TNF (clone MP6-XT22, Biolegend, San Diego, CA, USA). Data were collected on an LSR Fortessa flow cytometer (BD Biosciences, San Jose, CA, USA) and analyzed using FlowJo software v10.9 (Ashland, OR, USA) [22].

### 2.7. IFNγ-ELISpot Assay

Spleen samples from each subject group were processed to obtain and prepare single-cell suspensions. Cells were plated at 10^5^ cells/well in 96-well flat-bottom plates (Immobilon-P; Millipore, Billerica, MA, USA) that were pre-coated with anti-mouse IFNγ Ab (clone AN18; MABTech, Sweden) and incubated at 37 °C for 20 h with 10 μg/mL of SARS-CoV-2 peptides (NSP5_3420-28_- VSFCYMHHM; NSP6_3647-55_- VAYFNMVYM). IFNγ-secreting cells were detected using the Ready-SET-Go Kit (eBioscience, San Diego, CA, USA) according to the manufacturer’s instructions, and SFCs were counted using an ELISpot reader (MABTech, Nacka Strand, Sweden) [22].

### 2.8. Analysis of Cytokine Production by Cytometric Bead Array

Splenocytes obtained as previously described were plated at 10^5^ cells/well in 96-well flat-bottom plates and re-stimulated with antigen-specific SARS-CoV-2 peptides (NSP5_3420-28_- VSFCYMHHM; NSP6_3647-55_- VAYFNMVYM) for 24 h. The secretion of IFNγ, TNF, IL-6, and IL-10 was assessed using the Cytometric Bead Array Th1/Th2/Th17 kit (BD Biosciences, San Jose, CA, USA) following the manufacturer’s instructions. Samples were acquired using an LSR Fortessa flow cytometer (BD Biosciences, San Jose, CA, USA) and analyzed with FCAP Array 3 Software (BD Biosciences, San Jose, CA, USA).

### 2.9. CD4+/CD8+ T-Cell Depletion

Mice were injected intraperitoneally with CD4^+^ T-cell-depleting antibody (clone GK1.5, BioXCell, Lebanon, NH, USA), CD8^+^ T-cell-depleting antibody (clone 2.43, BioXCell, Lebanon, NH, USA), or an IgG2 isotype control antibody (clone LTF-2, BioXCell) at a dose of 250 μg intraperitoneally on days −3, −2, and −1 before the SARS-CoV-2 challenge. Blood samples were collected from each mouse before the SARS-CoV-2 challenge and analyzed via flow cytometry to confirm CD4^+^/CD8^+^ T-cell depletion [22].

### 2.10. Quantification of Viral RNA in Tissues

Organs were harvested and stored in a −80 °C freezer with TRIzol reagent (Thermo Fisher, Waltham, MA, USA). Defrosted tissues were homogenized at 30 Hz for 2 min using the Precellys 24 (Berkin), and then total RNA was extracted according to the manufacturer’s instructions. All RNA pellets were resuspended in 30 μg of RNase-free distilled water, quantified with a NanoDrop spectrophotometer (NanoDrop Technologies, Wilmington, DE, USA), and stored at −80 °C. The real-time reaction was conducted using the AgPath-ID™ One-Step RT–PCR kit reagents (Applied Biosystems, Foster City, CA, USA). SARS-CoV-2 RNA detection was performed according to the protocol described previously to amplify the SARS-CoV-2 E gene [22] (probe: FAM-ACACTAGCCATCCTTACTGCGCTTCG-BQ, primers: F: 5′ ACAGGTACGTTAATAGTTAATAGCGT 3′, R: 5′ ATATTGCAGCAGTACGCACACA 3′). Viral RNA concentration was calculated using a standard curve of four 100-fold serial dilutions of in vitro-transcribed RNA from SARS-CoV-2 (NC_045512.2_sars-cov-2_envelope).

### 2.11. Statistical Analysis

Data were analyzed using Prism software (GraphPad Software v9.1.1, San Diego, CA, USA) and presented as the mean ± SEM. Differences between group means were assessed with the Kruskal–Wallis test for more than two groups, or the nonparametric Mann–Whitney test for two groups. A *p*-value of <0.05 was deemed statistically significant. The Kruskal–Wallis test was used to analyze differences between group means.

## 3. Results

### 3.1. Design of Vaccine Constructs

**Figure 1:**
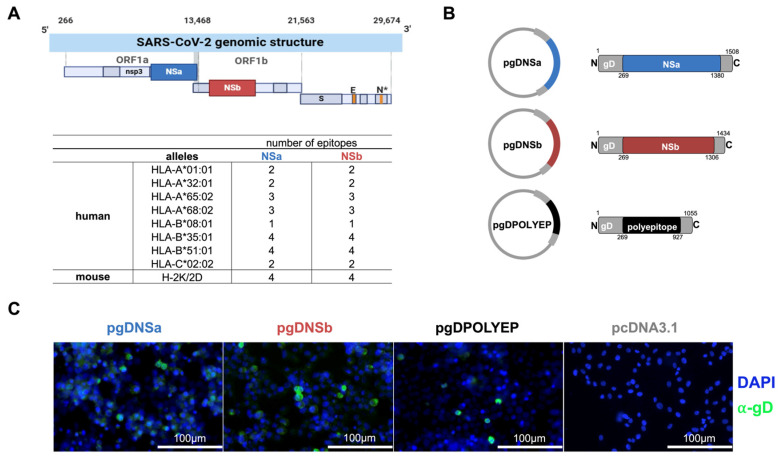
Schematic representation of epitope mapping for DNA vaccine design. (**A**) SARS-CoV-2 genomic structure highlighting regions with a high concentration of CD8^+^ T-cell epitopes recognized by the Brazilian population. HLA alleles were analyzed and identified, and epitopes were mapped to the NSa and NSb regions. (**B**) Schematic of gD-fusion proteins with residue numbering; insert regions are drawn to scale relative to gD (backbone not shown to scale). (**C**) Representative immunofluorescence images of HEK293-T cells transfected with the recombinant plasmids or the empty pCDNA3.1 vector and immunolabeled for glycoprotein D (green).

Through a comprehensive bioinformatic analysis [24], we identified two regions within the PR1ab region of the SARS-CoV-2 genome—designated NSa and NSb—that exhibited a high density of T-cell epitopes capable of binding a wide range of human and mouse MHC class I molecules (Figure 1A). Specifically, these regions contained multiple epitopes predicted to bind to common human HLA alleles, such as HLA-A01:01, HLA-A32:01, HLA-B35:01, and HLA-B51:01, which are prevalent among diverse populations, including the Brazilian population [25,26], ensuring broad immunological (T-cell) coverage and eight epitopes for the mouse H-2K/2D haplotype, allowing experimental in vivo testing of the vaccine-induced immunological and protective effects (Figure 1A).

The selected epitopes within NSa and NSb were sequentially linked using a GGGGS flexible amino acid linker [27], and then cloned into the pcDNA3.1 expression vector. The expressed sequence included a fusion to the glycoprotein D (gD) from Herpes Simplex Virus type 1 (HSV-1) to enhance immune responses [28,29]. The chimeric genes were under the control of a CMV promoter and a Kozak sequence for efficient antigen expression [30]. Three distinct vaccine constructs—pgDNSa, pgDNSb, and pgDPOLYEP—were created, with pgDPOLYEP incorporating all epitopes from both NSa and NSb regions fused to gD (Figure 1B).

To validate antigen expression, cells transfected with each construct underwent immunodetection of gD using FITC-labeled secondary antibodies. Immunofluorescence microscopy revealed the in vitro expression of the gD-fused antigens, indicated by pronounced green fluorescence signals, confirming the successful expression of the vaccine constructs (Figure 1C).

### 3.2. Evaluation of the Immunomodulatory Role of Glycoprotein D

**Figure 2:**
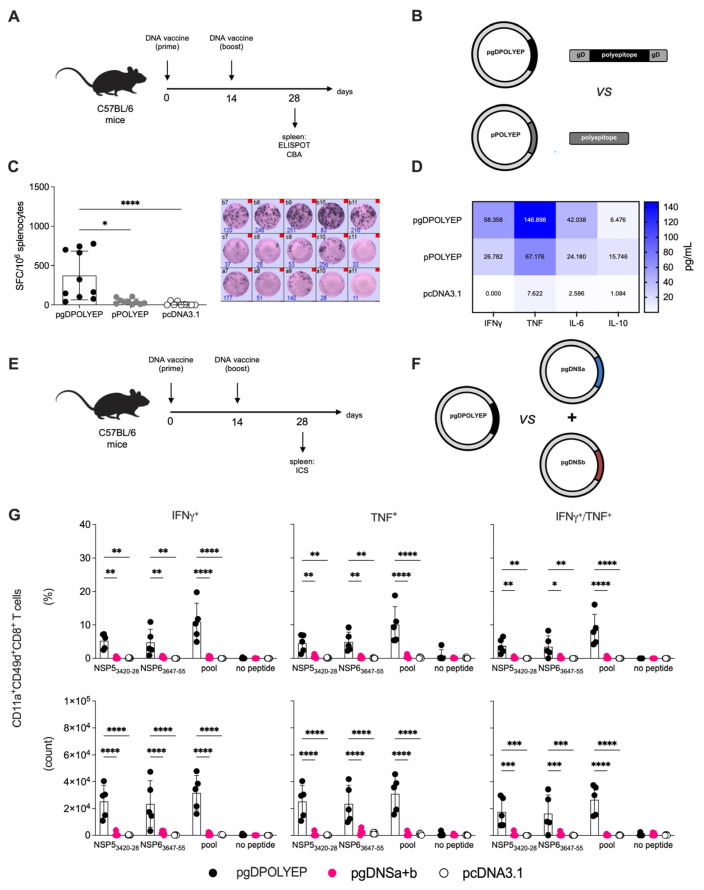
gD Fusion Boosts T-cell Immunogenicity of the Polyepitope DNA Vaccine. (**A**) C57BL/6 mice were immunized intramuscularly with two doses of (**B**) pgDPOLYEP or pPOLYEP, administered 14 days apart, followed by spleen collection on day 28. Splenocytes were stimulated for 24 h with SARS-CoV-2 H2-Db-restricted peptides. (**C**) ELISpot quantification of IFN-γ-producing spot-forming cells (SFC). Circles, individual mice; N = 10 mice/group (**D**) Cytometric bead array analysis of cytokine production in splenocytes culture supernatants. (**E**) C57BL/6 mice were immunized intramuscularly with two doses of (**F**) pgDPOLYEP, pgDNSa, or pgDNSb administered 14 days apart, followed by spleen collection on day 28. (**G**) ICS analysis of antigen-specific CD8+ T cells. Splenocytes were stimulated for 24 h with SARS-CoV-2 H2-Db-restricted peptides, immunolabeled for cell surface markers (CD11a+, CD49d+, CD8αlow), intracellular cytokines (IFN-γ, TNF), and analyzed by flow cytometry. Circles, individual mice; *n* = 5 mice/group. Data pooled from two independent experiments are presented as the mean  ±  SEM and compared by either the nonparametric Kruskal–Wallis test (**C**) or two-way ANOVA with Tukey’s multiple comparison test (**G**). * *p*  <  0.05; ** *p*  <  0.01; *** *p*  <  0.001; **** *p* < 0.0001.

The HSV-1 gD protein has been extensively studied for its immunostimulatory properties, especially in activating and expanding CD8+ T cells. Its ability to engage antigen-presenting cells and enhance T-cell priming has been applied in various vaccine platforms to strengthen immune responses [19,28,29]. To investigate this, we designed a comparative study using two vaccine constructs: (1) pgDPOLYEP, which includes the polyepitope sequence fused to gD, and (2) pPOLYEP, which retains the same polyepitope sequence but lacks the gD fusion. Additionally, the empty pcDNA3.1 vector served as a control. Six-week-old C57BL/6 mice were immunized intramuscularly via electroporation with two doses of either pgDPOLYEP or pPOLYEP, given 14 days apart (Figure 2A). Booster immunization was performed on day 14, and spleen collection was performed on day 28 to assess immune responses.

IFN-γ-secreting cells were quantified using the ELISpot assay after stimulation with SARS-CoV-2 H2-Db-restricted peptides (Figure 2B). ELISpot analysis showed a significant enhancement of the IFN-γ response in splenocytes from pgDPOLYEP-immunized mice compared to those receiving pPOLYEP or pcDNA3.1 (Figure 2C). Stimulation with a SARS-CoV-2 peptide pool induced a four-fold increase in IFN-γ-secreting cells in the pgDPOLYEP group relative to the pPOLYEP group (*p* < 0.001), suggesting that, indeed, gD functions as an effective immune enhancer of T-cell responses. The highest spot-forming cell (SFC) count was noted after stimulation with the peptide pool. In contrast, stimulation with individual NSP53420-28 and NSP63647-55 peptides generated lower but still significantly elevated responses compared to controls (*p* < 0.01) (Figure 2C). These findings confirmed that gD amplifies the magnitude of antigen-specific T-cell responses; therefore, subsequent experiments were carried out using the pgDPOLYEP vaccine construct.

To further characterize the induced immune responses, cytokine secretion was analyzed in culture supernatants collected from stimulated splenocytes (Figure 2D). The levels of IFN-γ, TNF, and IL-6 were significantly higher in the pgDPOLYEP group compared to the pPOLYEP and pcDNA3.1 groups (*p* < 0.001 for IFN-γ and TNF, *p* < 0.01 for IL-6). IL-10 secretion was minimal across all groups, suggesting that the vaccine formulation did not induce a regulatory immune environment. The elevated levels of pro-inflammatory cytokines indicate that the gD fusion enhances both the magnitude and quality of the immune responses by promoting a Th1-biased profile, which is crucial for effective viral clearance.

To specifically assess the functional CD8+ T-cell profile, C57BL/6 mice were immunized as previously mentioned; after the booster dose, the spleens of vaccinated mice were collected on day 28 and, after stimulation with SARS-CoV-2 H2-Db-restricted peptides (Figure 2E,F), intracellular cytokine production was determined by ICS, focusing on polyfunctionality (Figure 2G). Flow cytometry analysis of CD8+ T cells further confirmed the increased immunogenicity of the gD-containing vaccine (Figure 2E–G). The proportion of IFN-γ+ TNF+ double-producing CD8+ T cells was significantly higher in the pgDPOLYEP group compared to the pPOLYEP group (*p* < 0.001). Additionally, the frequency of antigen-experienced CD8+ T cells (CD49d+ CD11ahi CD8αlow) was notably higher in the gD-containing vaccine group (Figure 2G). These findings suggest that gD increases the number of antigen-specific CD8+ T cells and enhances their functional capacity.

These results demonstrate that incorporating HSV-1 gD into the polyepitope vaccine significantly enhances CD8+ T-cell-mediated immunity. The improvement is evident in the frequency and polyfunctionality of IFN-γ- and TNF-producing CD8+ T cells. Further studies were conducted to determine whether this enhanced response translates into superior protection upon viral challenge.

### 3.3. pgDPOLYEP DNA Vaccine Induced Short-Term Protection to SARS-CoV-2-Challenged K18-hACE2 Transgenic Mice

To further validate the protective efficacy of the pgDPOLYEP vaccine construct, we used the K18-hACE2 transgenic mouse model, which is a lethal model for SARS-CoV-2 infection (Wuhan strain). This model is advantageous because it transgenically expresses human ACE2 receptors in tissues, enabling efficient viral entry and replication, which can lead to disease [23,30].

**Figure 3:**
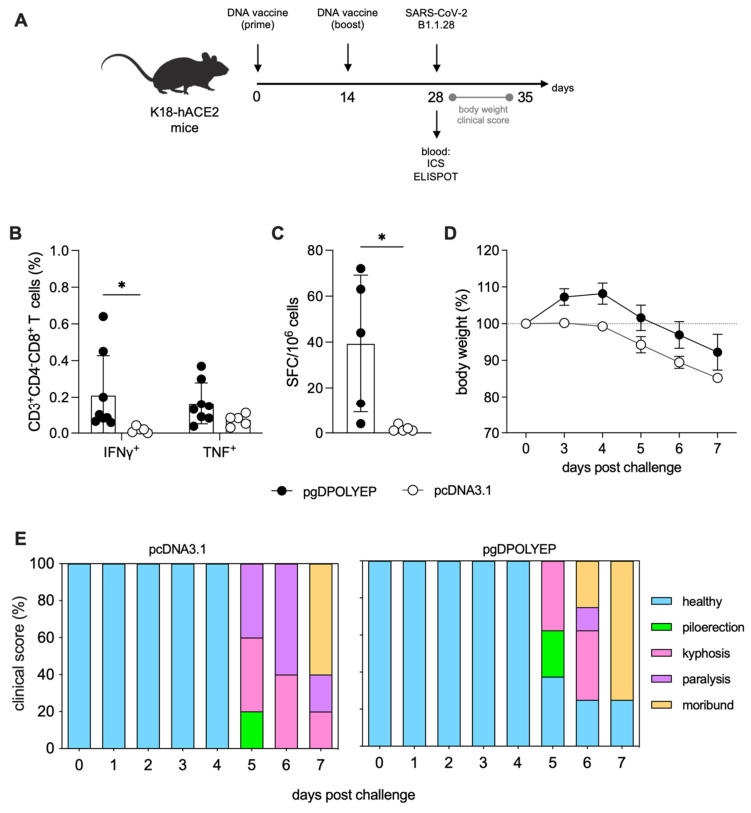
Immunogenicity and Protection by pgDPOLYEP in K18-hACE2 Mice. (**A**) K18-hACE2 mice were immunized intramuscularly with two doses of pgDPOLYEP, administered 14 days apart, followed by spleen collection on day 28. Mice were challenged with SARS-CoV-2 B.1.1.28 (5 × 10^4^ PFU, IN). (**B**) ICS analysis of antigen-specific CD8+ T cells on day 28. Blood cells were stimulated for four hours with SARS-CoV-2 H2-Db-restricted peptides and immunolabeled for cell surface markers (CD3+, CD4−, CD8+), intracellular cytokines (IFN-γ, TNF), and analyzed by flow cytometry. Circles, individual mice; N = 8 for pgDPOLYEP and N = 5 for pcDNA3.1 (**C**) ELISpot quantification of IFN-γ-producing SFCs in splenocytes isolated on day 28. Circles, individual mice; N = 5 for pgDPOLYEP and N = 5 for pcDNA3.1 (**D**) Body weight, (**E**) clinical score for signs of diseases were monitored for 7 days following the challenge on day 28. N = 8 for pgDPOLYEP and pcDNA3.1 groups (**D**,**E**). Data pooled from two independent experiments are presented as the mean ± SEM and compared by the Mann–Whitney test. * *p*  <  0.05.

K18-hACE2 mice were immunized intramuscularly using a standard protocol, which included electroporation, with two doses of the DNA vaccine (prime and boost) administered 14 days apart. Blood samples were collected two weeks after the second immunization to assess the induction of antigen-specific T-cell responses using intracellular cytokine staining (ICS) assays (Figure 3A). Analysis showed that the pgDPOLYEP vaccine induced significantly higher frequencies of CD3+CD8+ T cells, particularly those co-producing IFN-γ and TNF, compared to control mice immunized with the empty pcDNA3.1 vector (Figure 3B). These assays further supported ELISpot findings, demonstrating an increase in IFN-γ-secreting cell frequencies in spleens from pgDPOLYEP-vaccinated animals and robust antigen-specific T-cell induction (Figure 3C).

To assess the protective efficacy of the vaccine, immunized mice were intranasally challenged with 5 × 10^4^ PFU of the SARS-CoV-2 lineage B.1.1.28, following a previously established challenge protocol. Morbidity parameters, including clinical sign scoring and body weight changes, were monitored daily over seven days post-challenge. Control animals immunized with the empty pcDNA3.1 plasmid exhibited significant weight loss starting on day 3 post-challenge, dropping below 90% of their initial weight by day 6. In stark contrast, mice immunized with pgDPOLYEP exhibited stable body weight until day 3 after the challenge and showed significantly less weight loss thereafter, suggesting a decrease in disease severity (Figure 3D). Clinical symptom scoring, which included signs such as piloerection, kyphosis, and paralysis, further confirmed the protective advantage of the pgDPOLYEP vaccine. Vaccinated animals demonstrated lower symptom scores than controls, emphasizing the vaccine efficacy in mitigating morbidity (Figure 3E).

Collectively, these data confirm that pgDPOLYEP vaccination induces strong, multifunctional CD8+ T-cell responses and provides significant protection against SARS-CoV-2 infection, as evidenced by decreased morbidity parameters and sustained clinical health in the validated K18-hACE2 transgenic mouse model.

### 3.4. pgDPOLYEP Induces Durable T Cell Immunity and Enhanced Protection Against SARS-CoV-2 in K18-hACE2 Transgenic Mice

**Figure 4:**
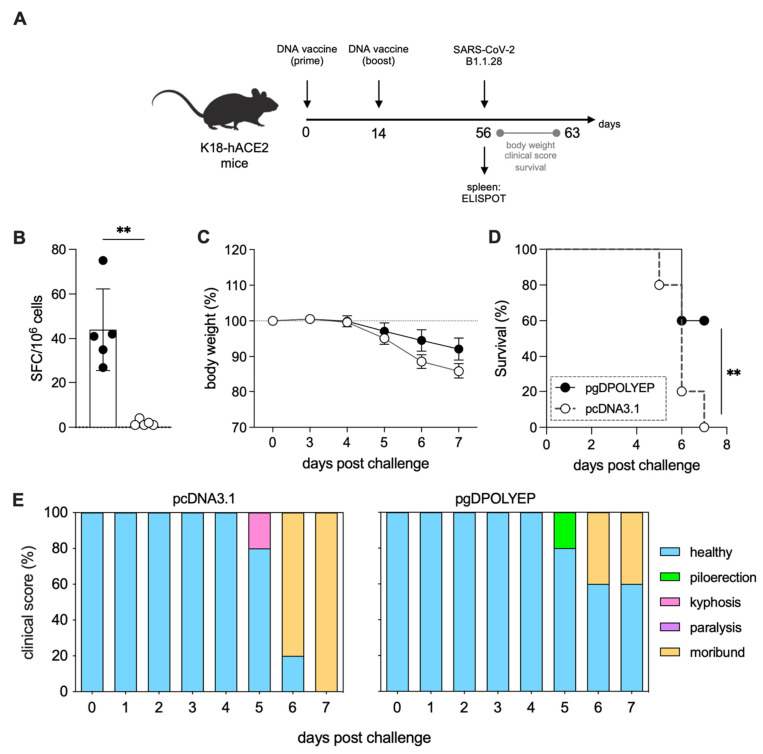
Memory-phase protection by pgDPOLYEP in K18-hACE2 mice. (**A**) K18-hACE2 mice were challenged 42 days post-booster (56 days post-prime); survival and weight loss were monitored through day 7 post-challenge, the acute disease window of this model. Mice were challenged with SARS-CoV-2 B.1.1.28 (5 × 10^4^ PFU, IN) on day 42. (**B**) ELISpot quantification of IFN-γ-producing SFCs in white blood cells collected on day 28, after four hours of stimulation with SARS-CoV-2 H2-Db-restricted peptides. Circles, individual mice; *n* = 5 mice/group (**C**) Body weight, (**D**) overall survival, and (**E**) clinical score were monitored for 7 days following the mice challenge on day 42. N = 10 for pgDPOLYEP and pcDNA3.1 groups (**C**–**E**). Data, pooled from two independent experiments, are presented as the mean ± SEM, and compared by the Mann–Whitney test (**B**), or the Mantel–Cox test (**D**), ** *p*  <  0.01.

Given the central role of sustained cellular immune responses in controlling SARS-CoV-2 infections, we evaluated the durability and protective efficacy of the immune responses elicited by pgDPOLYEP over an extended period. K18-hACE2 mice were immunized intramuscularly with two vaccine doses, each followed by electroporation (ipsilateral quadriceps for both doses), administered 14 days apart. To assess memory-phase immune persistence and protective efficacy, mice were intranasally challenged with SARS-CoV-2 on day 42 after the booster vaccination (Figure 4A). Antigen-specific T-cell responses were assessed using IFN-γ ELISpot assays on blood cells obtained on day 56 (42 days post-boost). The results showed significantly increased frequencies of IFN-γ-secreting T cells in the pgDPOLYEP-immunized mice compared to controls vaccinated with the empty pcDNA3.1 vector (*p* < 0.01) (Figure 4B).

Clinical protection was assessed by monitoring body weight and symptom severity following the challenge. Control mice immunized with the empty plasmid experienced rapid and severe weight loss, exceeding 20% by day 4 post-infection. In contrast, pgDPOLYEP-vaccinated mice maintained stable body weight up to day 3 post-infection and displayed significantly reduced weight loss during the acute disease window of the K18-hACE2 model (Figure 4C). Survival analysis revealed that pgDPOLYEP vaccination significantly improved survival outcomes, resulting in 60% survival at day 7 post-challenge, whereas control mice exhibited 0% survival. Because K18-hACE2 infection is typically lethal within ~6–8 days post-challenge, follow-up to day 7 captures the recognized acute phase in this model (Figure 4D,E). Clinical symptom scores further supported these findings, showing notably milder disease in vaccinated mice, characterized by fewer signs of morbidity such as piloerection, kyphosis, and paralysis (Figure 4E). Collectively, these data indicate that pgDPOLYEP elicits T-cell responses that persist through a delayed challenge and confer protection during the acute disease phase in K18-hACE2 mice.

### 3.5. T-Cells Are Critical for pgDPOLYEP Induced Protection to SARS-CoV-2 in K18-hACE2 Transgenic Mice

**Figure 5:**
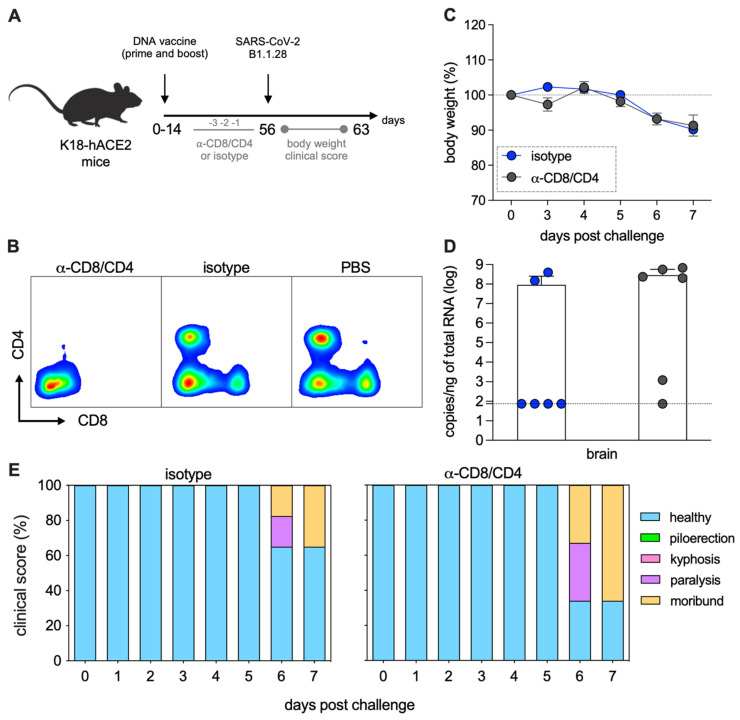
pgDPOLYEP-mediated protection requires T cells in K18-hACE2 mice. (**A**) K18-hACE2 mice were immunized intramuscularly with two doses of pgDPOLYEP, administered 14 days apart. Mice were challenged with SARS-CoV-2 B.1.1.28 (5 × 10^4^ PFU, IN) on day 42. (**B**) ELISpot quantification of IFN-γ-producing SFCs in white blood cells collected on day 28, after four hours of stimulation with SARS-CoV-2 H2-Db-restricted peptides. Circles, individual mice; *n* = 5 mice/group (**C**) Body weight, (**D**) overall survival, and (**E**) clinical score were monitored for 7 days following the challenge on day 42. *n* = 10 for pgDPOLYEP and pcDNA3.1 groups (**C**–**E**). Data pooled from two independent experiments are presented as the mean ± SEM, and compared by the Mann–Whitney test (**B**) or Mantel–Cox test (**D**).

To directly test the role of the T-cell compartment in the protective efficacy of the pgDPOLYEP vaccine, we conducted antibody-mediated depletion of CD4+ and CD8+ T cells before the viral challenge. K18-hACE2 mice immunized with the vaccine received anti-CD4 and/or anti-CD8 monoclonal antibodies intraperitoneally on days −3, −2, and −1 before challenge with SARS-CoV-2 (Figure 5A). The depletion efficiency was verified through flow cytometry of peripheral blood samples on day 0, showing nearly complete removal of the targeted CD4+ and CD8+ T-cell populations (Figure 5B).

Following the challenge, mice were closely monitored for indicators of morbidity, including body weight and clinical signs. Although peak body weight loss was similar across experimental groups, mice subjected to T-cell depletion exhibited more severe clinical symptoms, including pronounced piloerection, kyphosis, and paralysis, compared to isotype control-treated animals (Figure 5C–E). Notably, these clinical outcomes were associated with significantly elevated SARS-CoV-2 viral RNA levels in the brains of T-cell-depleted mice, as previously demonstrated [23], emphasizing the impaired control of viral dissemination in the absence of functional T-cell responses (Figure 5D).

## 4. Discussion

In this study, we introduced a polyepitope DNA vaccine that permitted us not only to demonstrate the ability of in silico-predicted antigens to generate robust immune responses in vivo but also to highlight the adjuvant effect of an embedded immunomodulatory domain, the Herpes Simplex Virus-1 glycoprotein D (gD), as an essential component driving T-cell-centric vaccines. This work provides a critical proof of concept that computationally chosen epitopes, when combined with a localized immune adjuvant, can induce functional immune responses capable of controlling SARS-CoV-2 infection in vivo.

A key innovation of our vaccine design lies in the strategic inclusion of the HSV-1 glycoprotein D. gD substantially enhanced vaccine immunogenicity, acting as a potent built-in adjuvant. This enhancement is primarily attributed to the interaction of gD with the herpesvirus entry mediator (HVEM), thereby counteracting inhibitory signals mediated by BTLA, a checkpoint receptor [31]. This targeted immune potentiation, conceptually analogous to localized immune checkpoint blockade, significantly amplified the magnitude and functional breadth of immune responses elicited by theoretically designed epitopes. Previous studies corroborate our findings, demonstrating that vaccines engineered to target the HVEM axis achieve markedly improved CD8+ T-cell responses and protective immunity against virus-induced conditions [18,28,29,30]. Notably, gD-enhanced responses do not require systemic adjuvants or broad immune activation, thus potentially minimizing off-target effects typically associated with traditional immunostimulatory agents.

This work also confirms the biological importance of computationally identified epitopes. Using in silico prediction tools, we assembled a variety of conserved epitopes targeting different SARS-CoV-2 structural and non-structural proteins, specifically designed for broad HLA presentation within the Brazilian population [32]. In addition, our results demonstrated that the selected epitopes induce measurable immune responses under in vivo conditions, providing essential experimental validation for our prediction pipeline. The immune response triggered by multiple antigens was associated with significantly lower viral loads and less severe disease in the K18 mouse model, supporting the potential of rationally designed epitope vaccines. Animals vaccinated with our formulation showed strong multi-antigen T-cell activation, especially against internal viral proteins that are less likely to mutate and evade immune detection [33]. Although T-cell-mediated immunity alone usually does not prevent initial infection at mucosal surfaces, it is a key factor for quickly controlling viral replication, reducing disease severity, and limiting transmission. Our findings are consistent with clinical reports that early, strong T-cell responses are linked to milder COVID-19 symptoms and faster viral clearance [34,35].

The targeting of conserved internal epitopes, rather than rapidly mutating surface epitopes, such as those in the spike protein, ensures that the vaccine-induced T-cell responses remain effective across emerging SARS-CoV-2 variants [33]. Recent clinical data affirm that T cells targeting internal viral antigens retain substantial reactivity (~80–90%) against highly mutated strains, in sharp contrast to neutralizing antibodies that frequently lose potency [36,37,38]. This quality positions our vaccine design as a potentially powerful supplement to antibody-focused vaccines, especially in scenarios involving immune-escape variants or in immunocompromised populations with diminished antibody responses [35]. Another notable strength of our platform is its adaptability. Epitope-based designs, combined with immune potentiators such as gD, offer inherent flexibility. Modular epitope arrangements enable rapid updates in response to emerging pathogens or new variants while maintaining broad HLA applicability, thereby streamlining vaccine redesign efforts [16].

It is necessary to recognize that while our vaccine construct demonstrates a protective effect, it remains a translational prototype. Protection primarily driven by cellular immunity generally results in non-sterilizing immunity; therefore, vaccinated individuals might still experience transient infection and viral shedding. Consequently, our vaccine approach could be most effectively used as a booster following traditional antibody-centric immunizations or specifically deployed among high-risk populations, such as immunosuppressed or elderly individuals, to enhance existing immune defenses. Still, by rapidly clearing infected cells, it could restrict viral replication, reduce transmission, and protect against pathology. This paradigm is analogous to observations in humans, where individuals with robust memory T cells often experience attenuated disease even if the infection is not completely prevented [34,35].

Although we did not perform head-to-head testing against authorized COVID-19 vaccines, relevant comparisons can be drawn. Licensed spike-centric platforms elicit strong neutralizing antibodies yet show diminished activity against antigenically drifted variants, highlighting vulnerability to immune escape [36,37,38]. By contrast, our polyepitope design targets conserved non-structural regions, and our findings are consistent with T-cell–directed vaccine studies that protect despite limited induction of virus-neutralizing antibodies, including a single-epitope peptide vaccine, a T-cell-biased spike vaccine, and an RBD–N chimeric protein in which protection mapped to cellular immunity [35,36,37]. Moreover, vaccine-induced T cells broadly cross-recognize variants [39,40,41], supporting the notion that our approach is complementary to antibody-forward formulations. Accordingly, a heterologous regimen—priming with an antibody-generating vaccine followed by a polyepitope T-cell boost—could combine durable neutralization with broad cellular memory [42,43,44,45]. Future work will optimize delivery and dosing and define the persistence and phenotype of memory T cells; notably, coronavirus-specific T-cell memory can be long-lived in humans [46].

Despite providing relevant proof-of-concept data for a novel polyepitope T-cell-targeted vaccine against SARS-CoV-2, our study has some limitations that warrant consideration. First, the vaccine efficacy assessments were performed exclusively using the SARS-CoV-2 B.1.1.28.2 lineage (Wuhan-related strain), and we did not directly evaluate protection against other variants of concern, such as Omicron or Delta. Since our vaccine design specifically targets conserved, non-spike epitopes predicted through computational analysis, we expect that the induced T-cell immunity will retain significant activity against diverse variants, as previously reported by multiple studies demonstrating cross-reactivity of T cells across SARS-CoV-2 variants due to epitope conservation [38,39]. Future studies directly challenging vaccinated animals with variant viruses will be crucial to validate this cross-protection hypothesis.

It is important to note that HLA genes are the most polymorphic in the human genome and encode molecules that play a central role in T-cell antigen presentation. Because our design targeted epitopes restricted to common Brazilian HLA alleles, we also included murine epitopes to experimentally validate immunogenicity and protective efficacy using the K18-hACE2 mouse model. This approach ensured rigorous preclinical testing while preserving translational relevance to human populations. Nevertheless, a limitation of our study is the absence of experiments employing HLA-transgenic or humanized mouse models, which would enable direct assessment of human epitope processing and presentation. Although our bioinformatic screening relied on prediction algorithms extensively validated against human T-cell epitope data [40,41], and the selected epitopes cover multiple common human HLA supertypes, validation in HLA-transgenic models should be prioritized in future studies to strengthen the evidence for clinical translation.

Although our study focused on intramuscular administration, alternative delivery routes may further enhance vaccine efficacy against respiratory pathogens. Intranasal vaccination has been shown to induce robust local B-cell memory and mucosal immune responses, including IgA production in the upper airways, which are less effectively generated by systemic vaccination [47]. These findings highlight the potential of mucosal vaccination strategies to complement or improve upon intramuscular delivery, particularly for pathogens such as SARS-CoV-2 that initiate infection at the respiratory mucosa. Future studies should evaluate whether our gD-fused polyepitope approach could similarly benefit from intranasal administration to elicit both systemic and local immunity.

## 5. Conclusions

Our study illustrates how combining computational antigen prediction with targeted immune potentiation via HSV gD results in a potent and versatile vaccine platform. By validating theoretically designed epitopes and demonstrating substantial in vivo protection, we pave the way for next-generation vaccine strategies capable of addressing evolving viral threats. This study highlights the potential of epitope-based, T-cell-centric vaccines enhanced by intrinsic immunomodulatory domains to serve as robust complements to traditional antibody-based approaches.

## 6. Patents

Patents related to the present work have been filed by LRMMA, BFMMP, RPSA, LRP, and LCSF, including BR102021006401-3, BR102022006089-4, and US#18/553,324. These patent applications cover key aspects of the technologies and methodologies described in this study.

## Data Availability

All data generated or analyzed during this study are included in the article. Additional information is available from the corresponding author upon reasonable request.

## Abbreviations

The following abbreviations are used in this manuscript:

ACE2	Angiotensin-Converting Enzyme 2
ANOVA	Analysis of Variance
APC	Allophycocyanin
BSA	Bovine Serum Albumin
BTLA	B and T Lymphocyte Attenuator
CD	Cluster of Differentiation
CEUA	Ethics Committee on Animal Use
CMV	Cytomegalovirus
COVID-19	Coronavirus Disease 2019
DC	Dendritic Cell
DMEM	Dulbecco’s Modified Eagle’s Medium
ELISpot	Enzyme-Linked ImmunoSpot
FACS	Fluorescence-Activated Cell Sorting
FBS	Fetal Bovine Serum
FITC	Fluorescein Isothiocyanate
gD	Glycoprotein D
HLA	Human Leukocyte Antigen
HSV-1	Herpes Simplex Virus type 1
HVEM	Herpesvirus Entry Mediator
ICS	Intracellular Cytokine Staining
IEDB	Immune Epitope Database
IFN-γ	Interferon Gamma
IgG	Immunoglobulin G
IL	Interleukin
IM	Intramuscular
IN	Intranasal
mRNA	Messenger Ribonucleic Acid
MHC	Major Histocompatibility Complex
N	Nucleocapsid Protein
NS	Non-Structural
ORF	Open Reading Frame
PBS	Phosphate-Buffered Saline
PFA	Paraformaldehyde
PFU	Plaque-Forming Unit
RNA	Ribonucleic Acid
RT–PCR	Reverse Transcription Polymerase Chain Reaction
S	Spike Protein
SARS-CoV-2	Severe Acute Respiratory Syndrome Coronavirus 2
SEM	Standard Error of the Mean
SFC	Spot-Forming Cell
Th1	T Helper type 1
TNF	Tumor Necrosis Factor
VOC	Variant of Concern
